# An incidental finding of testicular seminoma in the context of acute pulmonary embolism: a case report

**DOI:** 10.1186/s13256-021-02925-z

**Published:** 2021-07-20

**Authors:** Kaitlin J. Mayne, Emma Lewis, Lewis Vickers

**Affiliations:** University Hospital Wishaw, 50 Netherton Street, Wishaw, ML2 0DP UK

**Keywords:** Case report, Pulmonary embolism, Unprovoked, Cancer, Malignancy

## Abstract

**Background:**

Clinical guidelines do not recommend further investigation for occult malignancy in the scenario of unprovoked venous thromboembolism in the absence of additional clinical features suggestive of malignancy. We present the case of a young gentleman with pulmonary embolism who was diagnosed with testicular seminoma despite lack of symptoms or signs suggestive of malignancy. This is a unique case describing a scenario not well documented in existing literature where contravention of clinical guidelines had a potentially advantageous outcome for the patient.

**Case presentation:**

A 37-year-old white male presented with seemingly unprovoked acute pulmonary embolism with right heart strain. He did not have any predisposing factors for venous thromboembolism and did not have any symptoms or signs suggestive of malignancy. Clinical guidelines do not recommend further investigation to screen for malignancy in this scenario. Despite this, our young, otherwise healthy patient proceeded to computed tomography scanning, resulting in the diagnosis of localized testicular seminoma. Testicular ultrasound described normal-sized testes (despite a discrete lesion in the right testis), suggesting this was not detectable by the patient or clinician on routine examination. The patient was anticoagulated and had an inferior vena cava filter inserted to facilitate orchidectomy followed by adjuvant radiotherapy.

**Conclusions:**

This case highlights the importance of considering malignancy in seemingly unprovoked venous thromboembolism and the availability of guidelines to direct further investigation. Our patient’s treatment was not in line with clinical guidelines and was considered a “lucky find.”

## Background

Cancer is a well-established risk factor for venous thromboembolism (VTE) and, specifically, pulmonary embolism (PE). Studies have shown that 4–9% of patients diagnosed with apparently unprovoked VTE receive a cancer diagnosis within 12 months [1, 2]. Guidelines from multiple sources consistently recommend that further investigation for occult malignancy only be pursued when clinical assessment or routine investigation (chest x-ray, urinalysis) raises suspicion [3–6]. The British Society for Haematology recommends a lower threshold for further investigation in those over the age of 40 years or with bilateral deep vein thrombosis (DVT), early VTE recurrence, or a “very high” d-dimer result; however, “very high” is not quantified [4]. Additional investigations may result in more cancer diagnoses in patients with VTE compared with the recommended strategy of routine assessment and investigation; however, there is insufficient evidence to conclude whether this has any impact on mortality [1, 7, 8]. Cancer diagnosed in the context of VTE is often at an advanced stage at diagnosis, with one study quoting 12% 1-year survival in those with cancer at the time of VTE diagnosis [9]. For these reasons, in patients presenting with VTE with no features suggestive of malignancy in the clinical history and otherwise normal clinical examination and chest x-ray, further screening for malignancy is not recommended [3–5]. We believe this case is unique in that asymptomatic malignancy was identified in a patient presenting with pulmonary embolism in the absence of other clinical features and, therefore, in contradiction to clinical guidelines. Literature tends to focus on evidence-based practice; therefore, we feel there is value in sharing this case as an example of a scenario in which nonadherence to clinical guidelines may in fact have resulted in a beneficial outcome. We acknowledge that while the clinical decisions in this case resulted in earlier diagnosis of cancer, we cannot be sure this translates into survival benefit. Clinical guidelines are essential; however, we feel this case highlights the importance of synthesizing evidence with clinical judgment and making individualized clinical decisions.

## Case presentation

A 37-year-old white male presented to the Emergency Department with acute chest pain and breathlessness causing marked reduction in exercise tolerance. He denied loss of consciousness, cough, or other infective symptoms and had no symptoms of deep vein thrombosis. He did not have a personal or family history of venous thromboembolism nor any identifiable risk factors. He had been prescribed fluoxetine 60 mg once daily orally and promethazine 25 mg at night orally for low mood and insomnia and suffered mechanical back pain, but otherwise did not have any significant comorbidities. He is a non-smoker, drinks alcohol within recommended limits, denies recreational drug use, and works as a light technician.

On examination, the patient was febrile (temperature 37.8 °C), hypoxic (oxygen saturation 92% on room air), tachypneic (respiratory rate 24 breaths/minute), tachycardic (heart rate 120 beats/minute), and normotensive (blood pressure 132/84 mmHg). He was alert but appeared pale, with normal heart sounds and no murmurs heard. Auscultation of the chest was normal, and his abdomen was described as soft and nontender. There were no abnormalities on neurological examination. Electrocardiography (ECG) showed sinus tachycardia, right axis deviation, ischemic changes in the anterior leads, and the S1Q3T3 phenomenon.

Initial laboratory results are presented in Table 1. Assessment of d-dimer was not performed—we assume because clinical suspicion of VTE was sufficiently high to proceed directly to imaging and empirical treatment. Arterial blood gas sampling performed on room air revealed pO_2_ 7.6 kPa, pCO_2_ 7.6 kPa, hydrogen ion concentration 32 nmol/L, base excess 0.7 mmol/L, lactate 1.36 mmol/L, and oxygen saturation 92%. The patient routinely tested negative on severe acute respiratory syndrome coronavirus 2 (SARS-CoV-2) polymerase chain reaction (PCR) during admission, and there was no indication for other microbiological/serological testing.

**Table 1:** Laboratory results at presentation

Hemoglobin	15 g/dL	Sodium	145 mmol/L	Bilirubin	4 μmol/L
Total white blood cell count	16.9 × 10^9^/L	Potassium	3.7 mmol/L	Alanine aminotransferase (ALT)	40 U/L
Neutrophils	15.3 × 10^9^/L	Urea	5.9 mmol/L	Alkaline phosphatase (ALP)	107 U/L
Lymphocytes	0.8 × 10^9^/L	Creatinine	105 µmol/L	Albumin	45 g/L
Monocytes	0.8 × 10^9^/L	Chloride	106 mmol/L	C-reactive protein	45 mg/L
Platelet count	290 × 10^9^/L	Bicarbonate	24 mmol/L	Glucose	6.5 mmol/L
Prothrombin time	11.9 seconds	Estimated glomerular filtration rate (eGFR)	> 59 mL/minute/1.73 m^2^	Troponin T	374 ng/L
Activated partial thromboplastin time (APTT)	30.9 seconds				

The patient was treated for presumed pulmonary embolism with subcutaneous tinzaparin 17,000 IU/mL and proceeded to computed tomographic pulmonary angiography (CTPA). CTPA confirmed major pulmonary embolus, with associated acute right heart strain and dilation of the pulmonary trunk. The patient was transferred to the medical high-dependency unit for monitoring and continued treatment with tinzaparin. Echocardiogram confirmed the CTPA findings, demonstrating a dilated right heart with impaired right ventricular function and very mild tricuspid regurgitation.

The doctor in training responsible for his care at this point also requested a computed tomography (CT) scan of the abdomen and pelvis routinely to screen for malignancy. CT abdomen and pelvis was also documented in the management plan by two different consultant physicians. The same doctor in training then reviewed the patient 48 hours later and documented in the case notes at that time that National Institute for Health and Care Excellence (NICE) guidance dated March 2020 stated “not to offer further investigations for malignancy in patients with unprovoked PE unless relevant clinical signs or symptoms.” Despite this, the patient proceeded to CT scan without any symptoms or signs to suggest malignancy. The rationale for proceeding to CT scan is not documented in the medical notes and will be addressed in the discussion of the case.

**Fig. 1:**
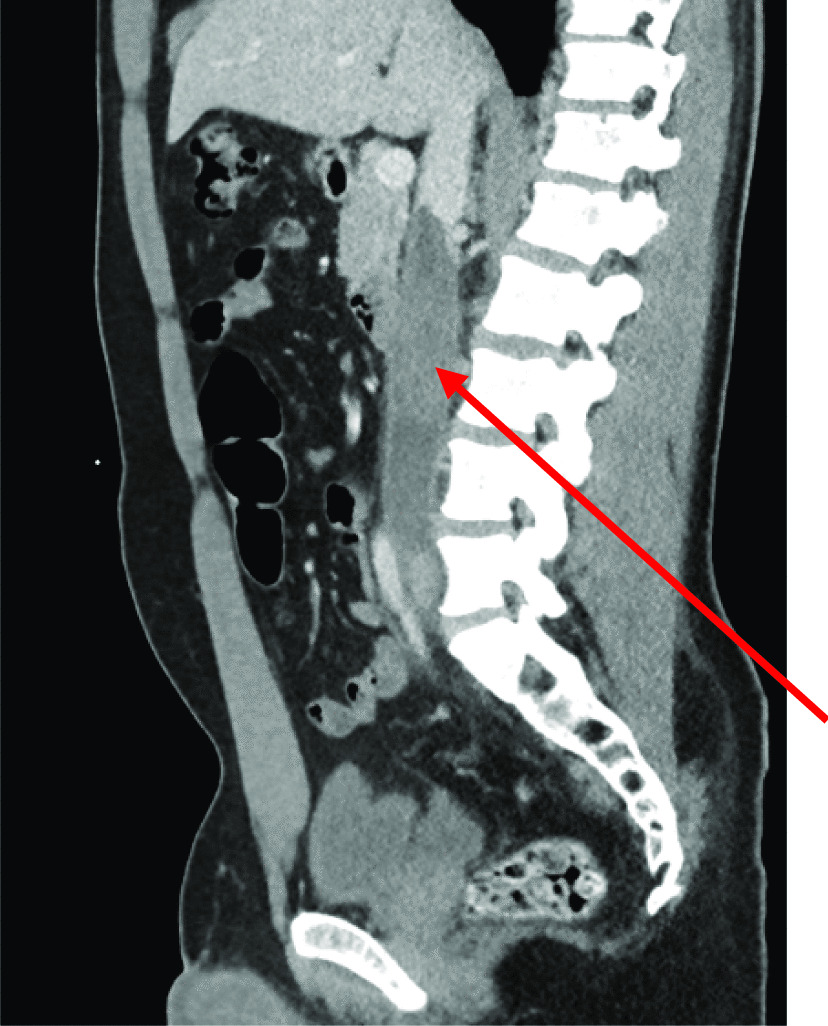
CT abdomen showing extensive thrombus (arrow) extending from the left common iliac vein through the IVC at the level of the renal veins

**Fig. 2:**
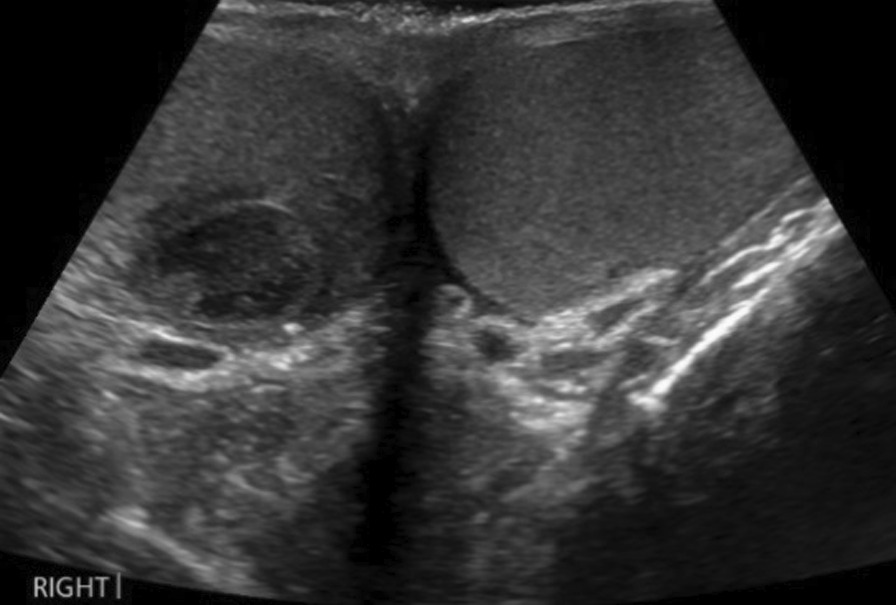
Testicular ultrasound showing hypoechoic lesion right testis and normal left testis

CT revealed extensive thrombus extending from the proximal left external iliac vein into the left common iliac vein and along the inferior vena cava (IVC) to the level of the renal veins (Fig. 1). The report also noted some prominent paraaortic nodes at the level of the renal veins and suggested these may be reactive. The solid abdominal organs appeared normal, and a small left-sided pleural effusion was noted. Due to unexplained lymphadenopathy, an astute clinician advised testicular examination. The patient did not self-examine regularly but denied testicular symptoms. Clinical examination was unremarkable, but he proceeded regardless to ultrasound examination. Ultrasound showed several hypoechoic and heterogeneous lesions identified within the right testis, the largest measuring 1.7 cm (Fig. 2). There was some vascularity within these lesions with appearances suggestive of testicular malignancy. The testicle did, however, appear normal in size, which would support the clinical examination findings.

Following discussion with the local Urology service, assessment of tumor markers was performed, which revealed elevated lactate dehydrogenase (LDH) at 449 U/L (reference range 0–250 U/L) with beta human chorionic gonadotropin (hCG) and alpha fetoprotein (AFP) within the normal range. The differential diagnosis was metastatic testicular cancer of germ cell origin or lymphoma. Tissue diagnosis would be required to confirm the diagnosis and guide treatment; however, orchidectomy was complicated by the need for anticoagulation in the context of significant thromboembolic disease.

Following multidisciplinary discussion, the patient was discharged to continue anticoagulant treatment with tinzaparin with a plan for delayed orchidectomy following reduction in clot burden. On the advice of the Haematology service, tinzaparin was changed to subcutaneous enoxaparin because of subtherapeutic anti-Xa levels following discharge. The dose was adjusted on several occasions; at the time of writing, the patient continues on enoxaparin 140 mg twice daily. Other medications administered were co-codamol 30/500 orally on an as-required basis for treatment of pain, allopurinol 300 mg once daily orally to reduce risk of spontaneous tumor lysis syndrome, and the patient’s preadmission medications: fluoxetine 60 mg once daily orally and promethazine 25 mg once daily orally, all of which were continued long term. The duration of anticoagulation had not yet been determined at time of discharge, pending cancer treatment and follow-up in due course.

**Fig. 3:**
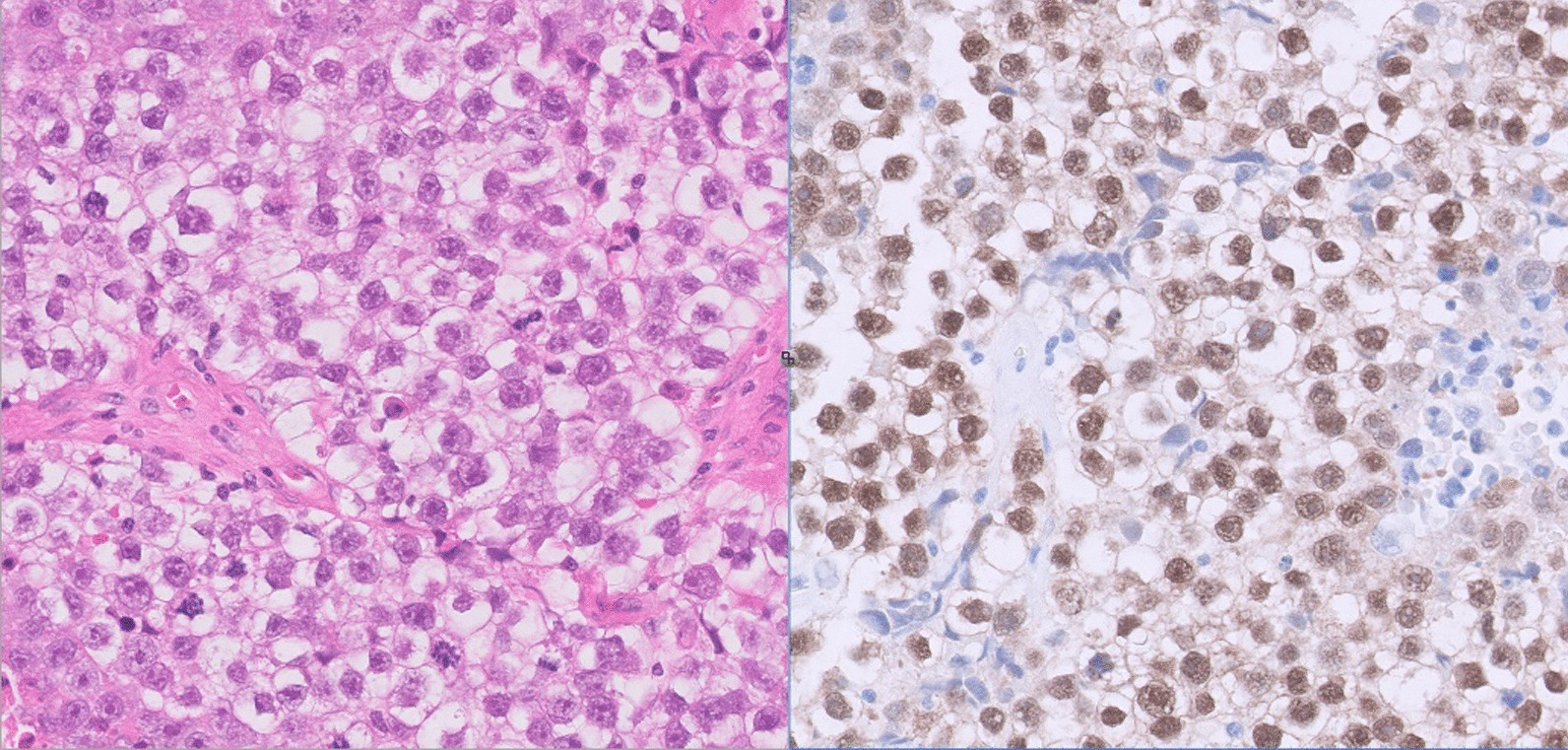
Tumor histology from the orchidectomy specimen consistent with seminoma on hematoxylin and eosin (H&E) staining (left). The image on the right shows positive immunohistochemistry for octamer-binding transcription factor (OCT) 3/4, a germ cell marker

Unfortunately, repeat imaging 20 days after presentation showed progression of the IVC thrombus and persistent pulmonary artery filling defects, corresponding to pulmonary embolism. IVC filter insertion was performed to facilitate right orchidectomy, which revealed 35-mm seminoma (Fig. 3) with invasion of the rete testis and hilar soft tissue.

Orchidectomy was complicated by scrotal hematoma, which was surgically evacuated. A repeat CT scan showed that aortocaval adenopathy had increased in volume from previous scans. Following multidisciplinary discussion, the patient’s disease was staged as stage 2A seminoma and the decision taken to proceed to adjuvant radiotherapy (30 Gray in 15 fractions) which was completed in December 2020. Less than 1 month later, abdominal CT imaging was repeated to assess burden of thrombus prior to IVC filter removal. This unfortunately showed multiple pulmonary and hepatic metastases. The decision was taken to proceed with bleomycin, etoposide and platinum (cisplatin) (BEP) chemotherapy, of which the patient completed four cycles in April 2021. Repeat CT showed good response to chemotherapy, and the patient remains well. He continues on therapeutic anticoagulation with enoxaparin and awaits specialist review by Haematology services to determine the duration of treatment.

## Discussion

It is unusual and not in line with clinical guidelines that this 37-year-old male with a first presentation of VTE and no clinical features of malignancy proceeded to CT scanning to screen for malignancy as a provoking factor for VTE. We feel this case highlights the importance of synthesizing evidence and clinical guidelines with clinical judgment and making individualized clinical decisions. Our patient had stage 2A seminoma at diagnosis and later developed metastatic disease. CT scan at the time of pulmonary embolism, although not indicated according to guidelines, resulted in earlier detection of cancer; however, we cannot be sure this translates into survival benefit.

Discussion surrounding this case revealed that some radiologists would have declined the initial CT request to screen for malignancy. The CT scan was requested by a doctor in training and featured again in subsequent consultant management plans. It had also been specifically documented that the clinical guidelines advised against the approach taken, and case note review did not reveal any specific suspicion of malignancy or documented indication for CT. We suggest the doctor in training felt obliged to follow instruction from senior clinicians and that this superseded guidelines. We suppose the consultant physicians involved either were not aware of the guideline or had sufficient suspicion of malignancy to proceed with CT scan regardless; however, we might expect the rationale to be documented in the latter scenario.

A meta-analysis found that, compared with routine investigation, more extensive investigation resulted in increased cancer detection initially but not by 12 months [1]. This included two large randomized-controlled trials of CT and 18F-deoxy-fluoro-glucose positron emission tomography (PET) in unprovoked VTE [10, 11]. It is recommended that patients diagnosed with PE are followed up with medical review at 3–6 months post-diagnosis to assess for VTE recurrence and bleeding complications of treatment and to determine duration of anticoagulation [5]. This review is also an opportunity to reassess for any provoking factors such as thrombophilia and signs or symptoms of malignancy that may not have been evident at time of VTE diagnosis [5]. Guidelines suggest that thrombophilia testing should not be offered in provoked VTE [5, 6]; therefore, there is no indication for testing in our patient with cancer as a provoking factor. Had this patient not been so extensively investigated, perhaps at 3–6 months follow-up there would have been clinically detectable signs of malignancy. It is not possible to predict the impact this would have on disease stage or amenability to treatment, but it is certainly possible earlier detection of cancer benefited our patient.

This case also draws attention to anticoagulation strategies in patients with cancer. In the absence of malignancy, the patient would have been treated with a direct-acting oral anticoagulant (DOAC) agent in line with current guidelines. Traditionally, patients with cancer are instead anticoagulated with low-molecular-weight heparin (LMWH), a treatment associated with greater cost and treatment burden. Patients with active cancer are known to have a greater risk of bleeding complications associated with anticoagulation [12]; therefore, the reversibility of LMWH may lead clinicians to favor this strategy. Early trials of DOACs included very low numbers of patients with cancer [5]; however, there is now a growing body of evidence to support the use of DOACs in patients with malignancy [13, 14]. 2019 European guidelines recommend that edoxaban or rivaroxaban should be considered (instead of LMWH), with the exception of patients with gastrointestinal malignancy (greater bleeding risk associated with DOACs [13, 14]) however, this is not yet reflected in clinical practice within the UK.

## Conclusions

The diagnosis of testicular seminoma in this case is considered a “lucky find,” and it is hoped that earlier detection and treatment may benefit the patient. We do not suggest, on the basis of this case, that clinicians disregard clinical guidelines and take the same investigative approach in every patient with a first presentation of VTE; to do this would result in harms due to unnecessary radiation exposure and would have resource implications for the health service. Clinical guidelines are based on critical appraisal of scientific evidence, weighing benefits against harms, and should be applied along with clinical judgment to inform evidence-based practice and ensure equality of patient care. This case highlights the importance of considering underlying malignancy in seemingly unprovoked VTE. Routine evaluation should take the form of clinical history, examination, urinalysis, and chest x-ray, with further investigation indicated only if initial evaluation raises concern.

## Data Availability

Data sharing is not applicable to this article as no datasets were generated or analyzed during the current study.

## Abbreviations

VTE: Venous thromboembolism
PE: Pulmonary embolism
DVT: Deep vein thrombosis
ECG: Electrocardiography
CTPA: Computed tomographic pulmonary angiography
CT: Computed tomography
NICE: National Institute for Health and Care Excellence
IVC: Inferior vena cava
LDH: Lactate dehydrogenase
hCG: Human chorionic gonadotropin
AFP: Alpha fetoprotein
PET: Positron emission tomography
DOAC: Direct-acting oral anticoagulant
LMWH: Low-molecular-weight heparin
